# Clock-Drawing Tasks as Predictive Measurements for Disease Classification Among Patients With Parkinson’s Disease and Essential Tremor

**DOI:** 10.7759/cureus.13239

**Published:** 2021-02-09

**Authors:** Anastasia M Bougea, Panagiotis Zikos, Ioanna Spanou, Efthymia Efthymiopoulou

**Affiliations:** 1 Neurology, Eginition Hospital, National and Kapodistrian University of Athens, Athens, GRC; 2 Neurology, 251 Hellenic Air Force Hospital, Athens, GRC

**Keywords:** parkinson’s disease (pd), essential tremor (et), clock drawing (cd) test, visuospatial and executive deficits

## Abstract

Background

Nonmotor cognitive symptoms are widely being recognized in both Parkinson’s Disease (PD) and Essential Tremor (ET), the two most common movement disorders. Clock-drawing (CD) test seems to be impaired early in the process of cognitive (executive) decline in PD. However, the optimal measures for detecting cognitive changes in ET patients have not been established. Examining whether the CD test is a quick test could identify frontal and visuospatial deficits in patients with Parkinson’s disease (PD) and essential tremor (ET).

Methods

Visuospatial performance was assessed in 58 consecutive patients with ET and 75 with PD and 22 healthy controls (HC) who visited two neurological clinics of Athens in Greece. The CD and copy (CC) items of the PD-Cognitive Rating Scale were used as a test of visuospatial function.

Results

Both CD and CC scores were lower for ET compared to PD patients and HC (p=<0.001 for both comparisons). A binomial logistic regression showed that both CD and CC items predict if participants had ET or PD with high sensitivity 94.7% and specificity 87.9% and an area under the curve (AUC) 0.980 (95% confidence interval, 0.962-0.997). The model explained 86.1% (Nagelkerke R2) of the variance in the disease variable (ET/PD) and correctly classified 91.7% of the cases.

Conclusion

Patients with ET have more visuospatial deficits compared to PD and HC. CD task may be an easy, useful tool to track cognitive changes in nondemented patients with ET in clinical practice.

## Introduction

In addition to motor features (tremor, bradykinesia), patients with Parkinson's disease (PD) also show a wide spectrum of nonmotor symptoms including cognitive deficits [1]. Approximately 20-40% of patients with PD progressively develop cognitive dysfunction and roughly 80% of patients with PD will have dementia after 20-25 years of disease [1]. However, cognitive dysfunction can occur in the early stages of PD and can present as a frontal dysexecutive syndrome [2].

Essential Tremor (ET) is defined as bilateral upper extremity action tremor for at least three years' duration, with or without tremor in other locations and no other neurological signs [3]. There is growing evidence that ET may be associated with cognitive deficits. Previous cross-sectional studies have shown that ET patients may have deficits in executive functioning, attention, concentration, verbal fluency, naming, recent and working memory [4-11]. However, the optimal measures for detecting cognitive changes in ET patients have not been established.

Clock-drawing (CD) is a rapid, inexpensive, and well-established screening instrument for dementia as a measure of spatial and executive dysfunction [12]. CD is used with high sensitivity and specificity for the detection of Alzheimer’s dementia (AD), but a poor sensitivity for the detection of early stage of AD [13]. By contrast, the CD test seems to be impaired quite early in the process of cognitive (visuospatial) decline in PD [10]. Furthermore, although there is great interest in the CD test as a cognitive screening tool, there are multiple CD administration and scoring systems with no consensus on which system produces the most valid results while remaining user friendly [14].

However, the above studies [4-11] have methodological limitations, including the absence of a control or PD group [6,8], no control for comorbidities [7], use of drugs that may affect cognition [6].

Thus, in light of the methodological limitations of previous studies and lack of optimal measures assessing cognition in patients with ET, the aim of this study was to examine whether the clock-drawing test as a quick and simple test could identify visuospatial deficits in patients with ET than those with PD.

## Materials and methods

This is a retrospective study of the clinical records (both written and electronic database) of 58 consecutive early, untreated (de novo) patients with ET, 75 with PD, and 22 healthy controls (HC) of similar age and education who visited two neurological clinics of Athens in Greece from January 1, 2018, to December 2019. 

Inclusion criteria were (1) age over 30 years (in order to avoid genetic/familial cases of PD with more early aggressive cognitive course); (2) ET and PD diagnosis recently made within the last five years, according to the most recent criteria [1,15]; (3) score 24 or more on Mini-Mental Status Examination (MMSE) cognitive screening instrument, (4) PD patients classified from 1 to 3 Hoehn and Yahr staging; (5) PD patients with regular prescriptions and in on phase of the medication; (6) visual acuity and sufficient hearing ability.

Exclusion criteria were (1) use of medications known to affect cognitive performance (primidone, topiramate, benzodiazepines, antidepressants, anticonvulsants, antihistamines, neuroleptics, and hypnotics); (2) major psychiatric disease or depression.

HC were recruited mainly from among patients’ relatives. Inclusion criteria for the HC were (1) the absence of a history or symptoms of tremor, PD, memory impairment, or other cognitive dysfunctions, and (2) absence of other neurological diseases, such as head trauma, epilepsy, and stroke, or brain surgery. The same exclusion criteria applied for ET and PD patients applied also for control subjects.

This study was approved by the local ethics committee, and each patient provided written informed consent to participate. 

All patients with ET were assessed using the Fahn-Tolosa-Marin Tremor Rating Scale for the severity of tremor [16]. The burden of PD was assessed by the Movement Disorders Society-United Parkinson’s Disease Rating Scale (MDS-UPDRS) part III (motor examination) in the ON medication state (e.g., pramipexol or selegiline or levodopa plus other drugs such as benserazide, pramipexol, or entacapone). Patients with ET continued the use of their tremor medication, if applicable, throughout the motor and cognitive evaluations. Levodopa equivalent dose (LED) was used to provide an equivalent oral Levodopa dose. The clock-drawing (CD) and copy (CC) items of the PD-Cognitive Rating Scale were used as a test of executive and visuospatial function [17]. In the CD task, participants were asked to draw a clock face on a blank sheet of paper, and to set the hands at “twenty-five minutes past ten” [17]. To assess the functionality of the posterior visual cortical areas, we assessed the copy of the presented clock after the unprompted drawing of such a clock, which has been shown to partially separate the frontal-subcortical from the posterior cortical component of this cognitive function [17]. The total score ranged from 0 to 10. These tasks do not interfere with motor abilities that could disadvantage PD or ET with moderate or severe tremors [18]. Cranial MRIs were notable for mild microvascular ischemic changes associated with normal aging, but not significant enough to account for their tremors, without cortical atrophy.

All data were analyzed using the Statistical Package for the Social Sciences (SPSS v. 3 22, IBM Corp., Armonk, NY, USA). Since the data did not meet the criteria for parametric statistical analysis, two independent-samples Kruskal-Wallis H tests were run, with adjusted a=0.025, to determine if there were differences in PD_ Umprompted drawing of a clock (UDC) and PD_DC scores between the three study groups. A receiver-operating characteristic curve (ROC) was performed to predict if participants had ET or PD.

## Results

The study population consisted of 155 subjects comprised 58 ET cases, 75 PD cases, and 22 HC. Demographic and clinical characteristics of ET, PD patients, and HC are shown in Table 1.

**Table 1:** Demographic and clinical characteristics of ET, PD patients, and HC HC: Healthy Controls; ET: Essential Tremor; LEDD: levodopa equivalent dose; MDS-UPDRS III: Movement Disorders Society-United Parkinson’s Disease Rating Scale; MMSE: Mini-Mental State Examination; PD: Parkinson’s Disease; PD_DC: PD_ Drawing Copy of a clock;  PD_UDC: PD_ Umprompted drawing of a clock; NS: not significant

	ET (n=58)	PD (n=75)	HC (n=22)	p-value
Age (mean±SD)	60.74± 8.4	61.04± 11	60.6 ± 7.45	NS
Gender (female/male)	24/34	45/30	11/11	NS
Education, years (mean±SD)	10.95±4.5	11.11±4.2	10.64 ±3.5	NS
Disease duration (years)	9.6±2.2	10.2±2.4	-	NS
Handedness R/L	35/23	52/23	16/6	NS
MDS-UPDRS III	-	26.6 ±12.4	-	
LEDD (mg/dl)	-	638±12.4	-	
MMSE (mean±SD)	27.2±2	26.6±2	-	NS
Clinic Rating Scale for Tremor	10±4	-	-	
PD_UDC (mean±SD)	6.55±2.01	8.65 ±0.6	9.65 ±0.57	<0.0001
PD_DC (mean±SD)	6.12 ± 0.92	8.93 ±0.25	9.74 ± 0.64	<0.0001

**Figure 1:**
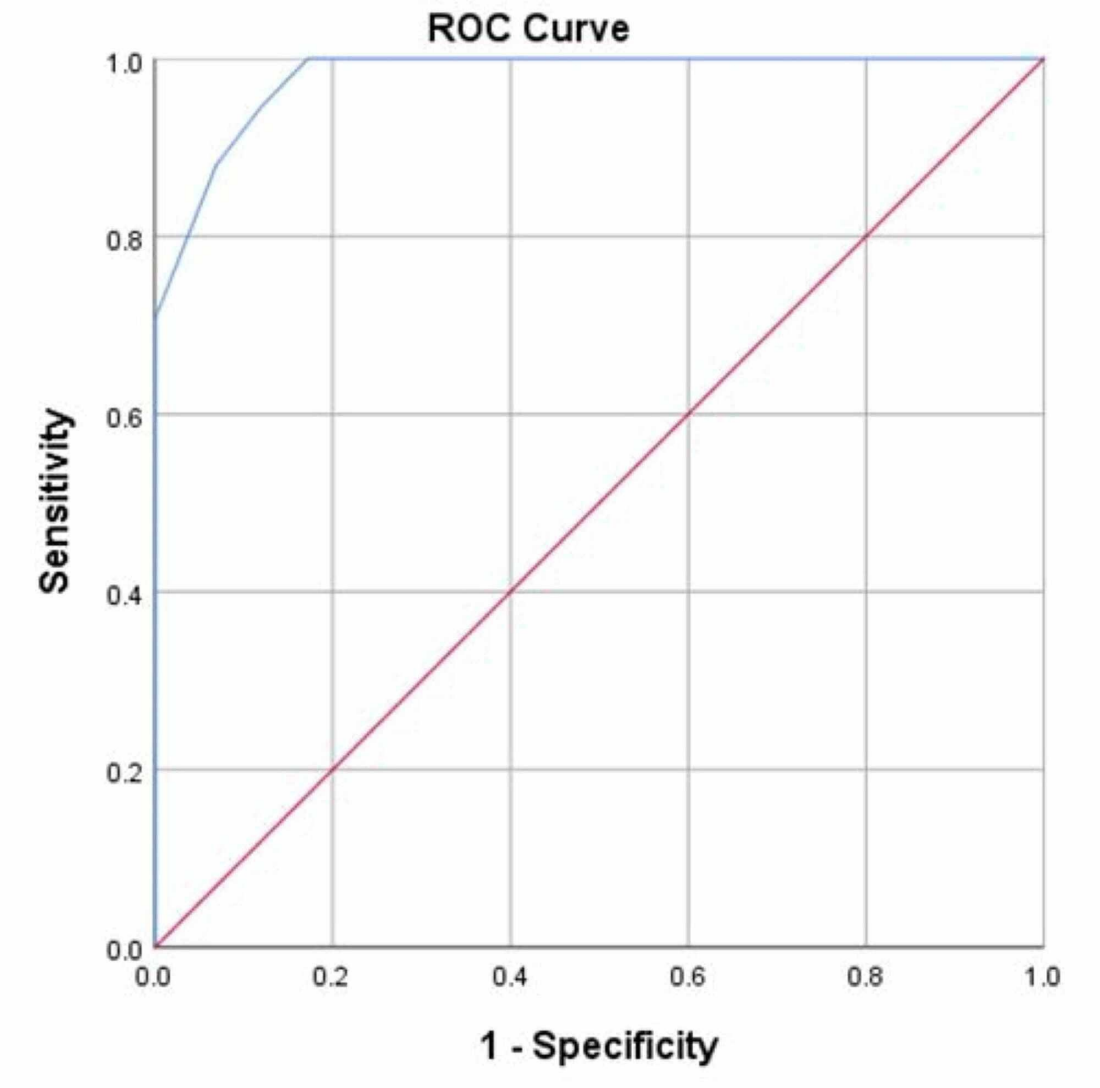
ROC curve to predict if participants had ET or PD ET: Essential Tremor; PD: Parkinson’s disease; ROC: Receiver-operating characteristic curve

There were no statistically significant differences between age, gender, education level, disease duration, and handedness of the patients with ET and comparison subjects. PD_UDC scores were higher, as expected, for HC compared to PD and ET patients, with those differences being statistically significant, χ^2^(2) = 125.606, p<0.001. PD_Copy drawing clock (DC) scores were also higher for HC compared to PD and ET patients, with differences reaching statistical significance, χ^2^(2) = 97.210, p<0.0001. A binomial logistic regression showed that both CD and CC items predict if participants had ET or PD with high sensitivity 94.7% and specificity 87.9% and an area under the curve (AUC) 0.980 (95% confidence interval, 0.962- 0.997) (Figure 1).

The model explained 86.1% (Nagelkerke *R*^2^) of the variance in the disease variable (ET/PD) and correctly classified 91.7% of the cases. Of note, there were no significant effects of tremor medications on the cognitive, or clinical variables of interest (all p>0.05).

## Discussion

Our novel findings are promising because CD test robustness lies in high sensitivity and specificity along with a high positive predictive value in a cohort of non-demented patients with ET.

The pathophysiology for the cognitive dysfunction (visuospatial) associated with ET is unclear, although a number of mechanisms may be responsible. Visuospatial disturbances in patients with ET may be due to the involvement of afferent pathways from the cerebellum to the posterior parietal lobe [19]. Moreover, while language and visual-spatial deficits in ET may be accounted for by disruptions in cerebellocortical networks, such deficits reflect local changes in the temporal and parietal cortex consistent with early AD (neuritic plaques, tau aggregates) [19,20]. Postmortem studies in ET cases have indicated the presence of brainstem Lewy bodies suggesting a link between ET and Lewy body disease [21]. Further prospective studies are needed to confirm whether ET is a slowly progressive neurodegenerative disorder.

So far, both ET and PD patients performed significantly worse on executive, auditory attention/working memory tests [22]. ET and PD exhibited similar deficits in attention, memory, language, executive/visuospatial function, particularly those thought to rely on the integrity of the prefrontal cortex, and this suggests the involvement of frontocerebellar circuits [23]. In accordance with our findings, these studies further challenge the traditional view of ET as a benign and monosymptomatic disorder.

Conversely, our results differ from those of Lombardi et al., who concluded that PD patients demonstrated poorer visuospatial skills (Facial Recognition Test) compared to ET [6]. Gasparini et al. reported that compared against ET patients, those with PD showed poorer performance in a number of verbal fluency and executive control tasks [9]. Benge et al. found that the executive control scale scores of 15 PD patients were inferior to those of 11 ET patients [18]. A recent functional fMRI study showed decreased functional connectivity in the visual and frontoparietal network of ET patients compared to HC [24]. It could be speculated that such functional changes in ET might represent an early marker of non-motor cognitive manifestations that have been related to ET. However, this possibility demands further investigation.

Another novel finding of this study is that a single clock-drawing test is a robust predictor of ET even after adjusting for age and education. Cersonsky et al. identified five non-motor tests in cognitive function domains that best predicted mild cognitive impairment in ET subjects [25]. High AUC scores among ET patients were observed for memory (86.2%) and executive control function (80.6%), but visuospatial ability in ET patients was not assessed. By contrast, our AUC scores were very high (98%) in predicting whether participants had ET or PD.

A particular strength of our study lies in the strict control of confounding factors such as tremor medications, depressive symptoms, and other neurological comorbidities as head trauma, epilepsy, and stroke, or brain surgery. In the Lombardi study [6], patients with ET were taking either propranolol or primidone; the latter is known to have potential negative cognitive effects [6,8], while other studies did not mention any tremor medications [4]. Whether neurological comorbidities affect the results is not yet clear, as relevant data are largely missing across the studies [4,6,9,11].

Despite our promising results, this study is not without its limitations. First, the retrospective design of the study. Second, our study included a relatively small sample size with a limited age range. Third, the MMSE, in fact, does not properly assess executive functioning, the most frequently and early impaired cognitive domain in PD. A condition of "early cognitive changes" could be diagnosed using different screening tools. The clock test can be an excellent screening method for these patients, but a complementary assessment, focused on executive function and spatial vision, would be important for better evaluation and interpretation of results. Nonetheless, we plan to expand our sample and assess the cognitive profile of these disorders longitudinally in a well-tolerated cognitive battery. 

This study also had several strengths. First, the study was population-based, allowing us to assess a group of patients with relatively mild ET compared to those with PD. Second, we performed a prediction model of both CD and CC items which correctly classified 91.7% of the cases (ET/PD). Fourth, we adjusted for the potential confounding effects of a number of important factors.

This article was presented as a poster at the 6th Congress of the European Academy of Neurology, Virtual 2020 in May 2020 [26]. 

## Conclusions

Although preliminary, the results of our study provide important new insights into the cognitive profile of ET and its underlying pathophysiology. These findings suggest that in clinical practice, the clock-drawing task could be an easy and useful screening tool in detecting early cognitive changes in non-demented patients with ET.
